# Protective role of female gender in programmed accelerated renal aging in the rat

**DOI:** 10.14814/phy2.12342

**Published:** 2015-04-22

**Authors:** Wioletta Pijacka, Bethan Clifford, Chantal Tilburgs, Jaap A Joles, Simon Langley-Evans, Sarah McMullen

**Affiliations:** 1 School of Physiology and Pharmacology, University of BristolBristol, UK; 2 Division of Nutritional Sciences, School of Biosciences, University of NottinghamLoughborough, UK; 3 Department of Nephrology and Hypertension, University Medical CentreUtrecht, The Netherlands

**Keywords:** Age, angiotensin II, angiotensin II receptor, estrogen, sex steroids

## Abstract

The aging kidney exhibits a progressive decline in glomerular filtration rate, accompanied by inflammatory and oxidative damage. We hypothesized that accelerated, age-related progression of renal injury is ovarian hormones-dependant. To address this we used an established model of developmentally programmed accelerated renal aging in the rat, superimposed by ovariectomy to assess interactions between ovarian hormones and the aging process. Under our experimental conditions, we found that kidney function worsens with age, that is GFR reduces over 18 month analyzed time-course and this was worsened by fetal exposure to maternal low-protein diet and absence of estrogen. Reduction in GFR was followed by increases in albuminuria, proteinuria, inflammatory markers, and tissue carbonyls, all suggesting inflammatory response and oxidative stress. This was associated with changes in AGTR2 expression which was greater at 18 months of age compared to earlier time points, but in MLP offspring only. Our studies show an influence of ovarian hormones on programmed accelerated renal aging and the AGTR2 across the lifespan. The main findings are that ovariectomy is a risk factor for increased aging-related renal injury and that this and oxidative damage might be related to changes in AGTR2 expression.

## Introduction

The aging kidney exhibits a progressive decline in glomerular filtration rate (Poggio et al. 2009; Rule et al. 2010), accompanied by inflammatory and oxidative damage, thickening of the glomerular basement membrane, and expansion of the glomerular mesangium and extracellular matrix (Zheng et al. 2004; Baylis 2005). The resultant glomerular sclerotic and ischemic injury leads to progressive loss of functioning nephrons, and is exacerbated by hypertension. Although the age-related decline in renal function may be sufficiently slow to have no obvious impact in healthy subjects, reduced renal reserve leaves the kidney susceptible to further damage when subject to additional insults. A preexisting nephron deficit, as a result of nutritional insults during development (Langley-Evans et al. 1999; Swali et al. 2011), could further accelerate the aging process.

Disturbance of the renin–angiotensin system (RAS) forms a central tenet of genetic, surgical, and diet-induced models of hypertension and declining renal function. Expression of angiotensin receptor type 2 (AGTR2) is upregulated in response to renal injury (McGrath and Welham 1999; Vazquez et al. 2005), promoting tissue remodeling via proliferation and apoptosis. Inhibition of AGTR2 following renal ablation potentiated subsequent development of hypertension, indicating a protective role. In contrast, however, other studies have shown AGTR2 to be involved in inflammatory cell recruitment (Hinton and Welham 1999).

We previously characterized expression of angiotensin II (Ang II) receptors in kidneys of rats exposed to a maternal low-protein diet or excess glucocorticoids during fetal life (McMullen et al. 2004; McMullen and Langley-Evans 2005b; a). Both protocols are associated with raised blood pressure and nephron deficits in adult animals. These studies showed significant sexual dimorphism in renal expression of AGTR2 suggesting that AGTR2 may promote compensatory renal function and tissue remodeling in response to the rising glomerular pressure associated with hypertension, but only in female offspring. Protective effects of female gender on progression of renal disease and hypertension has been demonstrated in many rodent models (Crofton et al. 1993; Vanhaesebroeck et al. 1999; Yin et al. 1999; Koeners et al. 2010). In humans, blood pressure is higher in men than in women (Baylis et al. 1988; August and Oparil 1999) and the incidence of hypertension is greater in men until women reach their seventh decade (Wyndham et al. 1987; Joles et al. 2010b), with men exhibiting a more rapid decline in renal function than women (Everitt et al. 1983). Protective effects of female gender may be mediated by interaction of estrogens with the renin–angiotensin and nitric oxide systems (Baiardi et al. 2005). We hypothesized that accelerated, age-related progression of renal injury is ovarian hormones-dependant. We also propose that the change in the AGTR2 protein expression might be associated with it. This investigation will provide basis for further analysis AGTR2 that might acts to ameliorate progression of renal injury and hypertension, and that this is an estrogen-dependent phenomenon.

To address this we used an established model of developmentally programmed accelerated renal aging (Langley-Evans et al. 1994, 1999; Joles et al. 2010a), superimposed by ovariectomy to assess interactions between estrogen and the aging process.

## Materials and Methods

### Experimental setup

#### Experimental design and generation of treatment groups

All experiments were carried out in accordance with the 1986 Animals (Scientific Procedures) Act. Forty-eight Virgin female Wistar rats (Harlan Ltd, UK) were mated at weights of 180–200 g. Upon confirmation of mating by the presence of a semen plug, rats were assigned to a control (CON, 18% casein) or a low-protein (MLP, 9% casein) diet as described previously (Langley and Jackson 1994; Joles et al. 2010a). Experimental diets were fed for the duration of pregnancy. At birth, all animals were transferred to a standard laboratory chow diet (B&K Universal Ltd, Hull, UK) and litters were reduced to a maximum of eight pups (four males and four females where possible) to minimize variation in nutrition during lactation. Offspring were weaned on to standard chow at 3 weeks of age and females were assigned to ovariectomy (OVX) or sham surgery (SHAM) at 10 weeks of age. Each litter was treated as a biological replicate. None of the females in the group were siblings. This generated four groups of 32 animals: CON-SHAM, CON-OVX, MLP-SHAM, and MLP-OVX. Eight animals from each group (based on power calculation) were selected for analyses at 6, 12, and 18 months of age. Each animal was killed at the assigned time point. The remaining eight rats per group were selected for telemetry surgery and cardiovascular monitoring at 12 months of age. Female littermates were randomly allocated to different groups to ensure that each treatment group at each time point was made up of nonsibling animals.

#### Sham and ovariectomy surgery and implantation of telemetry devices

All surgery was performed under isoflurane anesthesia in sterile conditions. Analgesia was administered as 0.005 mg/100 g of a semisynthetic opioid (Buprenorphine, Reckitt & Colman, Slough, UK) and 0.006 mg/100 g of a nonsteroidal antiinflammatory drug (Metacam, Boehringer Ingelheim, Germany) prior to surgery, under anesthesia following surgery and during recovery. At 10 weeks of age, all animals underwent bilateral ovariectomy or sham ovariectomy. Ovaries were removed through a single midline dorsal incision followed by bilateral muscle incision of 1 cm in maximum. Sham surgery involved the same incisions, but ovaries were left intact. A single stitch (Coated VICRYL, Johnson & Johnson Medical Ltd, Wokingham, UK) was applied to the muscle layer and running subcutaneous suture was used to close the skin incision. At 12 months, a subset of eight animals per treatment group had newly calibrated telemetry transmitters (PA-C40; DSI, St. Paul, MN) implanted into the abdominal aorta below the level of the renal arteries, as previously described (Joles et al. 2010b). Recordings were performed 1 week after full recovery via DSI Dataquest A.R.T.™ acquisition and analysis system. Systolic, diastolic blood pressures, and heart rate were collected over 24 h periods, continuously for five consecutive days.

#### End points: metabolic cages, euthanasia, and sample collection

As described above, eight nonsibling animals per group were sampled at each of the three time points. Animals were housed in metabolic caging for 24 h with free access to food and water, during which time urine was collected and food and water intake measured. After removal from metabolic cages, animals were killed by rising concentration of CO_2_ followed by cervical dislocation. Blood was collected by cardiac puncture into lithium-heparin microtubes (Sarstedt, Leicester, UK), centrifuged at 830 *g* for 10 min, and stored at −20°C prior to analysis. All major organs were dissected from the carcass, with kidneys separated into cortex and medulla. The wet weight of organs and gonadal and perirenal fat depots were recorded. Tissues were snap frozen in liquid nitrogen and stored at −80°C prior to analysis or fixed in 4% formalin.

### Biochemical assays

#### Plasma and urinary markers of renal function

All assays were performed using a microplate reader (Model 680 Microplate Reader, Bio-Rad Laboratories Ltd, Hemel Hempstead, UK). Plasma and urine creatinine were measured by the improved Jaffe method using a creatinine assay kit (Universal Biologicals, Cambridge, UK). Creatinine clearance was calculated from the standard formula: urinary creatinine [*μ*mol/L] × volume urine produced in 24 h [mL])/(plasma creatinine [*μ*mol/L] × 1440 [min] (Cornock et al. 2010). Urinary albumin was measured by the improved BCG method using an albumin assay kit (Randox Laboratories Ltd., Crumlin, UK). Total protein was measured by the Lowry method(Lowry et al. 1951) using a protein assay kit (Bio-Rad Laboratories Ltd, Hemel Hempstead, UK). Plasma urea was measured by the Jung method (Jung et al. 1975) using a urea assay kit.

#### Renal carbonyls

Kidney carbonyls were determined using the method described previously (Langley-Evans and Sculley 2005). Crushed, frozen kidney was homogenized in 50 mmol/L potassium phosphate buffer, 5 mmol/L EDTA, pH 7.4. Samples were assayed for protein content (mg/mL) DC protein assay kit (Bio-Rad Laboratories Ltd). Protein was precipitated from the samples by incubation with 500 *μ*L trichloroacteic acid (TCA) for 15 min at 4°C. Samples were then centrifuged at 15,588 *g* for 5 min, and the resultant pellet resuspended in either 2M hydrochloric acid (blanks), or 2M hydrochloric acid containing 0.1% 2,4-Dinitropheno (DNP). After incubating for an hour, protein was reprecipitated using TCA as before, centrifuged, and the pellet washed three times with ethanol:ethyl acetate solvent to remove excess DNP. The final pellet was suspended in 800 *μ*L of 6M guanidine hydrochloride and absorbance measured as 370 nm. The extinction coefficient of 21,000 mol/L per cm was then used to calculate the concentration of protein carbonyls in nmol/L per mg protein.

### Western blotting

**Figure 1:**
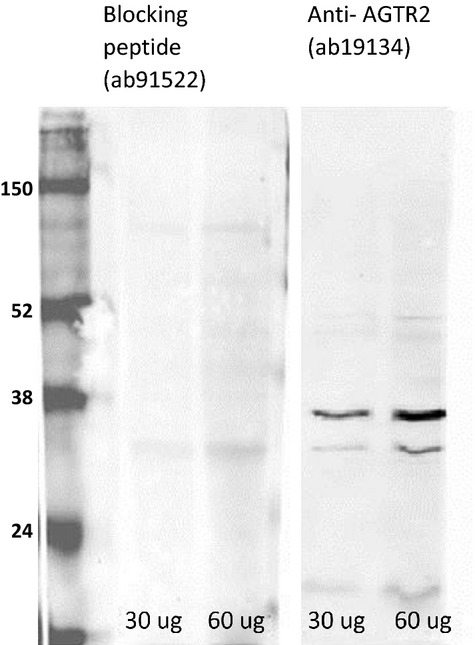
Representative western blot for AT2R to demonstrate antibody specificity. The incubation of the membrane with antibody neutralized by blocking peptide resulted in absence of band corresponding to the AGTR2 receptor. From the left: marker, 30 and 60 *μ*g protein sample incubated with blocking peptide/primary anti-AGTR2 antibody, then 30 and 60 *μ*g protein sample incubated with primary anti-AGTR2 antibody.

The right kidney cortex was crushed in liquid nitrogen with mortar and pestle and homogenized in protein extraction buffer (62.5 mmol/L Tris-HCl, 2% SDS, 5 mmol/L EDTA, 10% glycerol, pH 7.4). Homogenate was centrifuged at 10,000 *g* for 30 min at 4°C and the pellet discarded. Protein concentration of the supernatant was determined by the Lowry method using a protein assay kit (Bio-Rad Laboratories Ltd). The Amersham ECL Plex Western blotting system using Low-fluorescent PVDF membrane (GE Healthcare, Buckinghamshire, UK) and two CyDyes (GE Healthcare), the Cy3 and Cy5 coupled to secondary antibodies, were used for all analyses. This system enables detection and quantification of two different proteins on the same membrane with broad dynamic range and high linearity. Total protein of 60 *μ*g was used for protein electrophoresis. After protein transfer, the membrane was blocked for 1 h at room temperature with 2% Advance blocking agent (GE Healthcare). Anti-AGTR2 (ab19134, Abcam) and nuclear factor *kappa*-light-chain-enhancer of activated *B* cells (NF- *κ*B, sc-7151, Insight Biotechnology Limited, Middlesex, UK) at dilutions of 1:2,000 and 1:100, respectively, were incubated with membrane over night at 4°C. The membranes were subsequently washed with 1xPBS/0.1%Tris three times for 5 min at room temperature. Anti-Histone H2B (ab52484) primary antibody at 1:40,000 dilution incubation with membrane for 1 h at RT was followed by washing and then incubation with secondary antibody. Incubation with secondary antibody diluted in wash buffer (Cy3 anti-mouse, 1:4000 and Cy5 anti-rabbit, 1:3000) was carried out in the dark at RT. Before imaging membrane was thoroughly washed and dried in order to reduce the background. The secondary antibody signal was detected by scanning the membrane using a fluorescent laser scanner, Typhoon (GE Healthcare). Image was quantified by the ImageQuant (GE Healthcare). The optimal primary and secondary antibody concentrations were determined by performing a test on the different protein amount and different dilutions. Each gel included an internal control sample to allow standardization between blots. Specificity of AGTR2 antibody was confirmed by blocking peptide: AGTR2-ab91522 (Abcam), Figure1.

### Histochemistry

**Figure 2:**
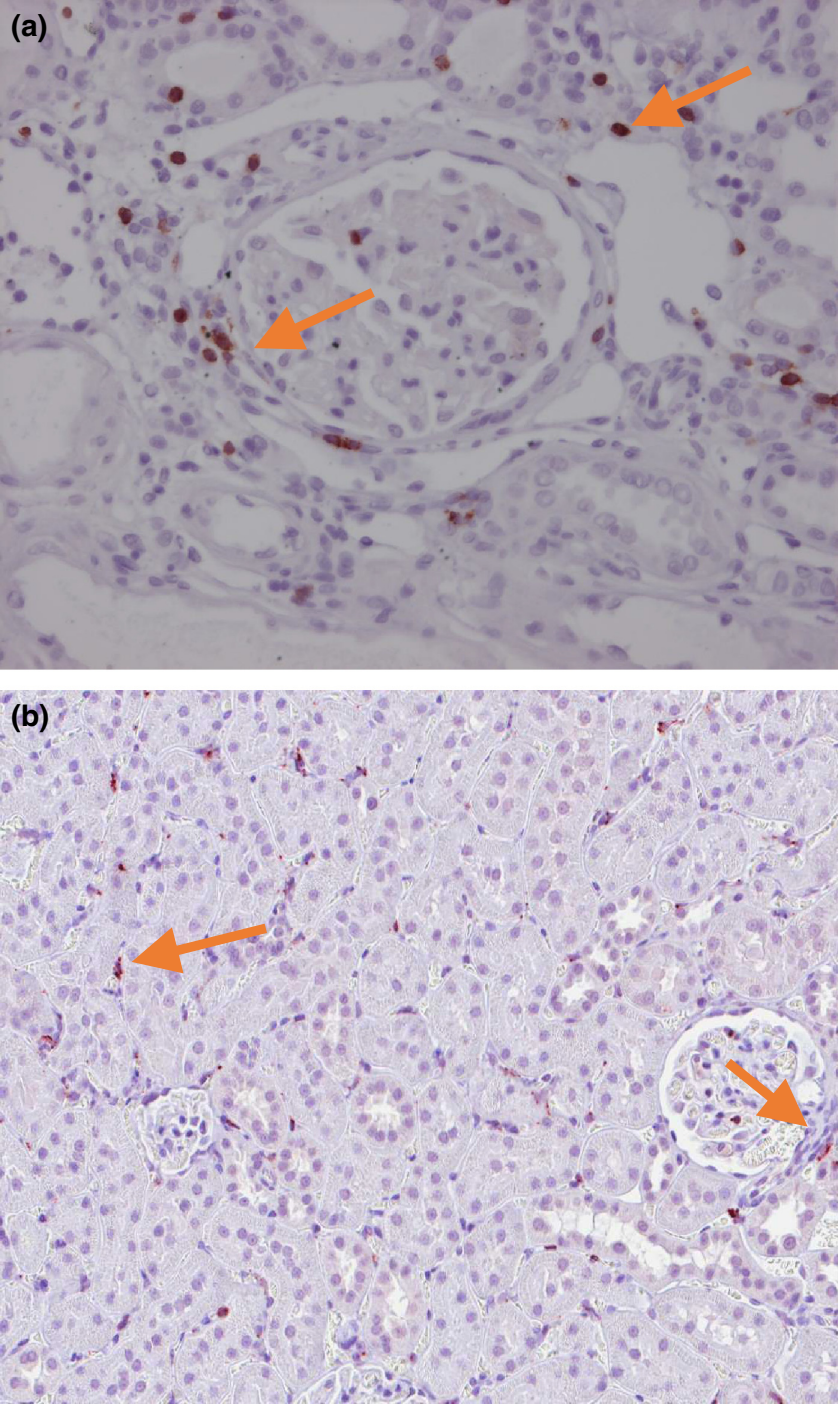
Representative images of CD3 and ED1 positive cells in left kidney. The image A corresponds to the CD3 and image B corresponds to the ED1. Arrows indicate examples of positive staining.

Left kidneys were fixed in 4% Formalin/1xPBS pH 7.4 (Sigma-Aldrich Company Ltd, Dorset, UK) for 24–48 h at room temperature and then sequentially dehydrated in 50% and 70% ethanol, prior to being processed/embedded in paraffin using a tissue processor (Histokinette Benchtop, Fullerton, CA, USA). Fixed kidney samples were processed and embedded in paraffin using a tissue processor (Histokinette Benchtop). Macrophages were stained with an antibody to ED1 as described (Attia et al. 2002). Lymphocytes were stained with an antibody to CD3 as described (Bongartz et al. 2012). Brightvision-HRP was used as secondary antibody (Immunologic, Tilburg, the Netherlands). Positive cells were visualized with Vector Nova Red (Vector) and counterstained with hematoxilin. ED1 and CD3 positive cells were counted in left kidney. In kidney sections, positive cells in 50 glomeruli and 20 peritubular areas were counted (magnification 400×) (Fig.2).

### Statistical analysis

The main and interactive effects of age, maternal diet and offspring ovariectomy on kidney function, inflammatory responses, protein expression, and blood pressure were analyzed by 3-way analysis of variance (ANOVA) using SPSS (IBM, NY). A post hoc Bonferroni test was applied where applicable. All data are presented as mean ± standard error of the mean (SEM). *P* < 0.05 was accepted as statistically significant in all analyses.

## Results

### Body weight, fat deposition, and organ weights

Body weight increased across each time point, independently of diet and surgery (main effect of age, 6 < 12 < 18, *P* < 0.001, Table1). There was no effect of maternal diet on body weight, but ovariectomy increased body weight relative to sham controls at all time-points (SHAM<OVX, *P* < 0.001). When expressed as a % of body weight, the weight of the gonadal (6 < 12&18, *P* < 0.001) and perirenal (6&12 < 18, *P* < 0.001) fat depots increased with age (Table1). However, the effect of age on the weight of the gonadal fat depot was modified by ovariectomy (age*surgery interaction, *P* < 0.01). This reflected a more rapid increase in gonadal fat deposition in OVX compared to SHAM animals, which then plateaued after 12 months. Ovariectomy increased the weight of the peri-renal fat deposit across all ages, independently of age (SHAM<OVX, *P* < 0.001).

**Table 1:** Body and organ weights of female offspring at 6, 12, and 18 months of age

Offspring weights	Diet	6 months		12 months		18 months		Three-way ANOVA
		SHAM Mean ± SEM	OVX Mean ± SEM	SHAM Mean ± SEM	OVX Mean ± SEM	SHAM Mean ± SEM	OVX Mean ± SEM	
Body weight (BW, g)	CON	264.7 ± 7.8	287.8 ± 8.4	295.9 ± 15.1	350.6 ± 15.1	372.4 ± 13.8	422.1 ± 13.8	6 < 12 < 18 *P* < 0.001 SHAM<OVX *P* < 0.001
	MLP	273.3 ± 7.8	330.2 ± 7.8	290.5 ± 13.1	370.9 ± 13.1	366.5 ± 13.8	429.6 ± 13.8	
Gonadal fat (% of BW)	CON	2.43 ± 0.29	3.11 ± 0.31	3.14 ± 0.32	3.84 ± 0.35	3.97 ± 0.35	3.92 ± 0.35	6 < 12, 18 *P* < 0.001 SHAM<OVX *P* < 0.001 Age^*^Surgery *P* < 0.01
	MLP	2.41 ± 0.29	2.83 ± 0.29	2.57 ± 0.30	4.37 ± 0.28	4.25 ± 0.35	4.03 ± 0.35	
Perirenal (fat % of BW)	CON	1.99 ± 0.22	2.70 ± 0.24	2.50 ± 0.45	3.78 ± 0.39	2.93 ± 0.33	3.72 ± 0.33	6 < 12 < 18 *P* < 0.001 SHAM<OVX *P* < 0.001
	MLP	1.54 ± 0.24	2.31 ± 0.24	1.81 ± 0.34	3.46 ± 0.34	2.62 ± 0.33	4.61 ± 0.33	
Left kidney (% of BW)	CON	0.30 ± 0.01	0.24 ± 0.01	0.33 ± 0.01	0.27 ± 0.01	0.28 ± 0.01	0.22 ± 0.01	6, 12 < 18 *P* < 0.001 SHAM>OVX *P* < 0.001
	MLP	0.29 ± 0.01	0.26 ± 0.01	0.31 ± 0.01	0.22 ± 0.01	0.27 ± 0.01	0.22 ± 0.01	
Right kidney (% of BW)	CON	0.33 ± 0.02	0.24 ± 0.02	0.36 ± 0.02	0.26 ± 0.01	0.28 ± 0.01	0.23 ± 0.01	6, 12 < 18 *P* < 0.001 SHAM>OVX *P* < 0.001
	MLP	0.29 ± 0.02	0.25 ± 0.02	0.36 ± 0.01	0.23 ± 0.01	0.28 ± 0.01	0.23 ± 0.01	
Liver (% of BW)	CON	3.61 ± 0.13	2.51 ± 0.13	3.16 ± 0.11	2.43 ± 0.12	2.85 ± 0.12	2.21 ± 0.12	6 > 12 > 18 *P* < 0.001 SHAM>OVX *P* < 0.001
	MLP	3.51 ± 0.13	2.89 ± 0.13	3.31 ± 0.09	2.40 ± 0.09	2.65 ± 0.12	2.30 ± 0.12	
Lungs (% of BW)	CON	0.54 ± 0.03	0.46 ± 0.03	0.57 ± 0.03	0.48 ± 0.03	0.56 ± 0.06	0.41 ± 0.06	SHAM>OVX *P* < 0.001
	MLP	0.59 ± 0.03	0.54 ± 0.03	0.56 ± 0.02	0.45 ± 0.02	0.53 ± 0.06	0.55 ± 0.06	
Heart (% of BW)	CON	0.41 ± 0.02	0.31 ± 0.02	0.32 ± 0.02	0.26 ± 0.02	0.30 ± 0.01	0.25 ± 0.01	6 > 12, 18 *P* < 0.001 SHAM>OVX *P* < 0.001 Diet^*^Surgery *P *< 0.05
	MLP	0.34 ± 0.02	0.33 ± 0.02	0.32 ± 0.01	0.27 ± 0.01	0.30 ± 0.01	0.26 ± 0.01	

Animals were exposed to a prenatal control (CON) or low-protein (MLP) diet, with sham or ovariectomy (OVX) surgery at 10 weeks of postnatal age. Organ weights are expressed as a percentage of body weight. Data are presented as mean ± S.E.M. Three-way ANOVA assessed the main factor effects of age, diet, and ovariectomy, with post hoc analysis where effects of age were significant. Where there was a significant interaction of diet or ovariectomy with age, the data were split by age for two-way ANOVA to assess the main factor effects of diet and ovariectomy at each time point (NS: non significant, ^*^denotes interaction).

Organ weights generally reduced as a percentage of body weight with age (kidneys: 6&12 > 18, *P* < 0.001; liver: 6 > 12 > 18, *P* < 0.001: lungs: NS, heart: 6 > 12&18, *P* < 0.001, Table1). Analysis of absolute organ weights (data not shown) suggested that this reflected increased fat deposition rather than a reduction in organ weights. Ovariectomy resulted in decrease of absolute weight of right (SHAM 0.97 ± 0.02 vs. OVX 0.88 ± 0.02) and left (SHAM 0.91 ± 0.02 vs. OVX 0.85 ± 0.02) kidneys and the liver (SHAM 9.6 ± 0.2 vs. OVX 8.8 ± 0.2), *P* < 0.001.

### Renal function

**Figure 3:**
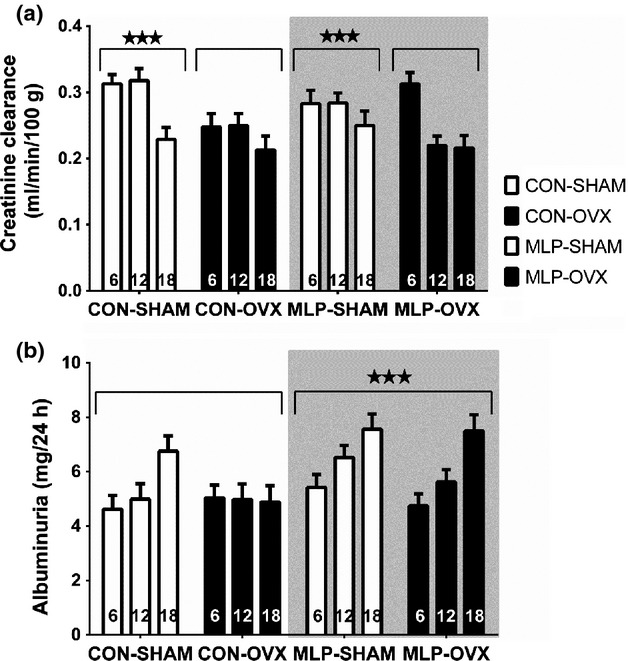
Urinary albumin concentrations and creatinine clearance as a proxy for glomerular filtration rate (GFR) in female offspring at 6, 12, and 18 months of age. Animals were exposed to a prenatal control (CON) or low-protein (MLP) diet, with sham (SHAM) or ovariectomy (OVX), (SHAM-open bars, OVX – closed bars), surgery at 10 weeks of postnatal age. Data are presented as mean ± SEM for *n* = 8 per group. Three-way ANOVA showed a significant main factor effects of age and prenatal diet for albuminuria and ovariectomy and age for creatinine clearance. (*P* < 0.001***, *P* < 0.01**, *P* < 0.05*)

Creatinine clearance (mL/min/100 g BW) was higher in intact females across the whole data set and it decreased with age (main factor effect of ovariectomy, SHAM>OVX, *P* < 0.001 and age, 6 > 12 > 18, *P* < 0.05, Fig.3). Increasing age was associated with an increase in plasma urea in control offspring only (age*diet interaction, *P* < 0.05, Table2) and an increase in urinary albumin between 12 and 18 months of age in all groups (main effect of age, *P* < 0.001). Urinary albumin and protein were higher in MLP offspring relative to controls across all ages (main effect of diet, *P* < 0.001 for both), particularly at 18 months (Fig.3). Urine volume was greater in MLP offspring (main effect of diet, *P* < 0.01) and this was associated with greater 24 h water intake (main effect of diet, *P* < 0.05). The effects of ovariectomy on measures of renal function all acted independently of age or maternal diet; main effects of surgery are therefore reported. OVX animals exhibited reduced urine volume (*P* < 0.05) which were associated with reduced water intake (*P* < 0.001, Table2).

**Table 2:** Markers of renal function in female offspring at 6, 12, and 18 months of age

Marker	Diet	6 months		12 months		18 months		Three-way ANOVA
		SHAM Mean ± SEM	OVX Mean ± SEM	SHAM Mean ± SEM	OVX Mean ± SEM	SHAM Mean ± SEM	OVX Mean ± SEM	
Plasma urea (mmol/L)	CON	7.2 ± 0.4	7.7 ± 0.4	8.5 ± 0.5	8.5 ± 0.5	8.2 ± 0.3	7.2 ± 0.3	12 > 18 *P* < 0.05 Age^*^Diet *P *<* *0.05
	MLP	9.0 ± 0.4	7.7 ± 0.4	8.4 ± 0.5	8.5 ± 0.5	7.1 ± 0.3	7.0 ± 0.3	
Urine protein (mg/24 h)	CON	75.9 ± 4.3	71.5 ± 4.1	63.2 ± 5.1	64.4 ± 5.6	79.5 ± 6.1	63.7 ± 6.1	6 > 12 < 18 *P* < 0.001 CON<MLP *P* < 0.001
	MLP	76.5 ± 4.1	82.7 ± 4.1	73.2 ± 4.4	66.5 ± 4.4	97.1 ± 6.1	85.7 ± 6.1	
Urine volume (mL/24 h)	CON	11.4 ± 1.6	10.0 ± 1.5	14.6 ± 1.9	9.1 ± 2.1	11.4 ± 1.3	7.4 ± 1.1	CON<MLP *P* < 0.01 SHAM>OVX *P* < 0.05
	MLP	13.2 ± 1.4	13.9 ± 1.8	15.8 ± 1.8	7.4 ± 1.9	14.3 ± 1.2	12.0 ± 1.2	
Water intake (mL/24 h)	CON	27.5 ± 2.7	18.3 ± 2.9	26.1 ± 2.8	20.9 ± 2.5	24.9 ± 1.8	18.9 ± 1.8	CON<MLP *P* < 0.05 SHAM>OVX *P* < 0.001
	MLP	27.8 ± 2.7	25.9 ± 2.7	32.3 ± 1.9	19.9 ± 2.1	25.0 ± 1.9	25.1 ± 1.8	
Food intake (g/24 h)	CON	22.1 ± 1.5	19.6 ± 1.5	21.7 ± 1.6	20.4 ± 1.6	21.1 ± 1.5	15.4 ± 1.5	NS
	MLP	20.7 ± 1.5	22.7 ± 1.5	20.6 ± 1.4	20.8 ± 1.4	22.0 ± 1.5	19.9 ± 1.5	

Animals were exposed to a prenatal control (CON) or low-protein (MLP) diet, with sham or ovariectomy (OVX) surgery at 10 weeks of postnatal age. Data are presented at mean ± SEM. Three-way ANOVA was assessed the main factor effects of age, diet, and ovariectomy, with post hoc analysis where effects of age were significant. Where there was a significant interaction of diet or ovariectomy with age, the data were split by age for two-way ANOVA to assess the main factor effects of diet and ovariectomy at each time point (NS: non significant, ^*^denotes interaction).

### Blood pressure and heart rate analysis

**Figure 4:**
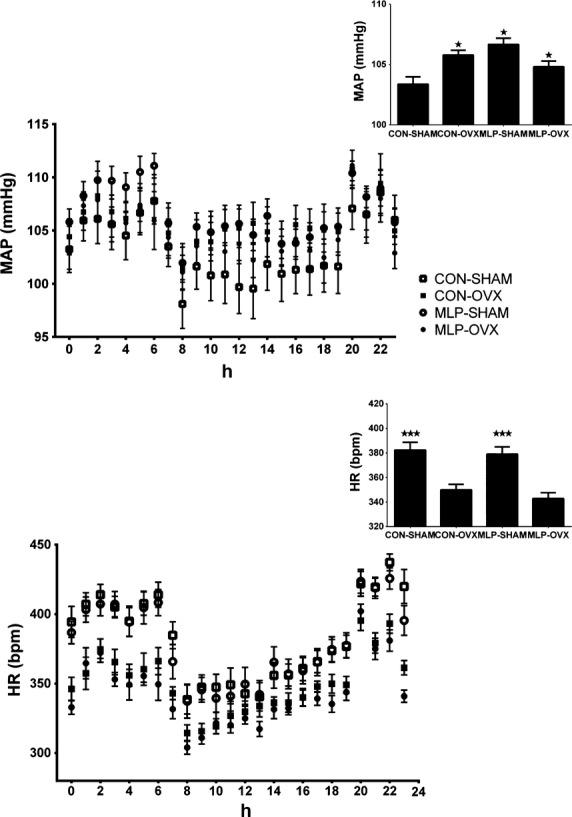
Mean arterial blood pressure (MAP) and heart rate (HR) in control (CON) and low-protein (MLP) intact (SHAM) and ovariectomized (OVX) offspring at 12 months of age. Data were collected continuously over a 5 day period by radiotelemetry and means calculated to give a 24 h profile. A two-way ANOVA looking at the diet and ovariectomy effects showed that MAP was the lowest in the CON-SHAM during light cycle, (*P < 0.05 compared to CON-SHAM) and there was a tendency for it to be lower over full 24 h period, *P* = 0.06. HR was significantly increased in OVX group (***P < 0.001 compared to OVX of same dietary background).

Blood pressure was determined by radiotelemetry in offspring at 12 months of age and we observed an interaction between maternal diet and offspring ovariectomy in their effects on resting mean arterial blood pressure (MAP), (*P* < 0.05). MAP was the lowest in the CON-SHAM during the light period and there was a tendency to be lower over full 24 h period, although this did not achieve statistical significance (*P* = 0.06, Fig.4). There was no effect of maternal diet on heart rate. However, ovariectomized females exhibited lower heart rate compared to intact females, (*P* < 0.001, Fig.4).

### Protein AGTR2 expression and protein carbonyls

**Figure 5:**
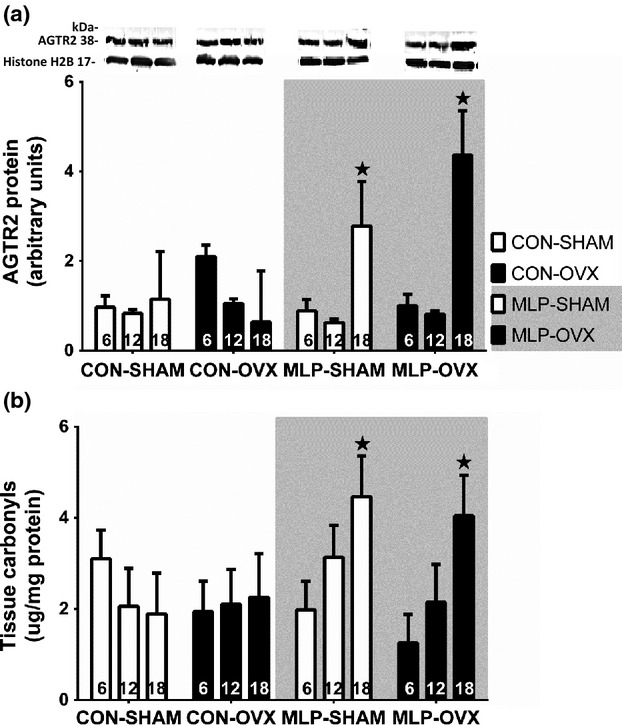
Western blot analysis and cropped images of the protein expression of angiotensin receptor type 2 (AGTR2) and the protein carbonyls concentration in the renal cortex at 6, 12, and 18 months of age. AGTR2 expression increased at 18 months of age, due to increases in MLP groups (*P* < 0.05*). Three-way ANOVA for AGTR2 showed a significant interaction between the diet and age, (*P* < 0.05*). The tissue carbonyls increased with age, due to increases in MLP groups 6 < 18, (*P* < 0.05*) and there was an interaction age*diet, showing an increase in tissue carbonyls at 18 month in MLP group, (*P* < 0.05*). White columns represent sham females (SHAM-open bars) and black columns represent ovariectomized females (OVX-closed bars). Data are presented as mean ± SEM for *n* = 8 per group.

Expression of AGTR2 was determined by western blot to assess the hypothesis that this protein was responsive to effects of both maternal diet and ovariectomy. There was interaction between effects of diet and age in their effects on AGTR2 protein expression (age*diet interaction, *P* < 0.001, Fig.5). At 18 months of age, expression was greater than at earlier time points (age, *P* < 0.05) but this age-related effect was only observed in the MLP-exposed animals. Tissue protein carbonyl content was measured as a marker of oxidative damage. There was evidence of greater damage in MLP offspring but only at 18 months of age (diet*age interaction, *P* < 0.001).

### NF-*κ*B protein expression and inflammatory changes in the kidney

**Figure 6:**
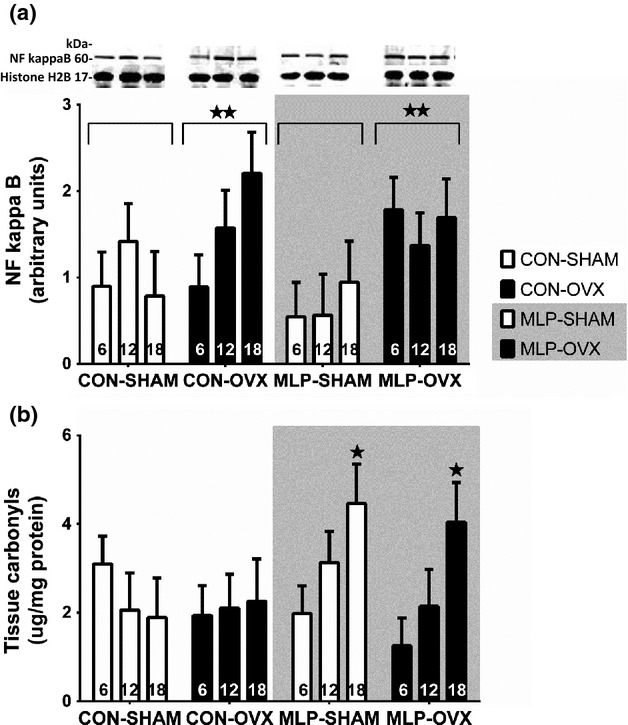
Western blot analysis and cropped images of NF-*κ*B protein expression in the renal cortex at 6, 12, and 18 months and positive cell counts for CD3 marker in glomerulus collected at 6, 12, and 18 months. Animals were exposed to a control (CON) or low-protein (MLP) diet during pregnancy, with sham or ovariectomy (OVX) surgery at 10 weeks of postnatal age. White columns represent intact (SHAM) females and black columns represent ovariectomized (OVX) females. Data are presented as mean ± SEM for *n* = 8 in each group. There was a significant main factor effect of ovariectomy in increasing NF-*κ*B protein expression (*P* < 0.005). Main factor effects of age, due to decreases after 6 months (*P* < 0.001***) and ovariectomy (OVX>SHAM, *P* < 0.05*) observed for CD.

Expression of NF-*κ*B protein, which is a transcription factor for proinflammatory genes including cytokines, chemokines, and adhesion molecules (Fig.6) has been measured in the renal cortex. The expression of NF-*κ*B protein was not affected by age. Ovariectomy significantly increased the expression of NF-*κ*B protein in the renal cortex (*P* < 0.005). There was no effect of maternal diet. Histochemical analysis of sectioned kidneys showed that glomerular infiltration by CD3^+^ cells (Fig.6) and ED1^+^ cells was higher at 6 months of age compared to 12 and 18 months (6 months: 1.20 ± 0.05, 12 months: 0.48 ± 0.06, 18 months: 0.52 ± 0.06; *P* < 0.001). OVX animals had a higher number of CD3^+^ cells compared to SHAM, independently of age or maternal diet (*P* < 0.05). There were no effects of age, diet or surgery on inflammatory markers in the tubulointerstitial region (data not shown).

## Discussion

It has been shown that maternal protein restriction impairs nephrogenesis and programs hypertension in rats (Langley-Evans et al. 1999; Schreuder and Nauta 2007; Bongartz et al. 2010) and that elements of the mechanism which drives the long-term response to maternal undernutrition are sex-specific. We hypothesized that accelerated, age-related progression of renal injury is ovarian hormones-dependant. We also propose that the change in the AGTR2 protein expression might be linked with it. Estrogen has been associated with increased resistance to hypertension and slower kidney aging in female subjects (Bongartz et al. 2010). Our studies show the influence of ovariectomy on renal aging and the renin–angiotensin system across the lifespan. The main findings of this study are that absence of female hormones is a risk factor for increased aging-related renal injury and that this is related to changes in expression of AGTR2.

Ovariectomy performed at 10 weeks of age resulted in visible and easily measurable changes in the body weights (Table1) and gonadal, and perirenal fat deposition. Ovariectomized females were clearly heavier than intact females and therefore showed lower organs weight in relation to the total body mass (Table1). Our data are in agreement with other studies showing increase in the body weight post ovariectomy. Studies on the rodent model for human menopause suggest that body weight is mediated by the estrogen receptor alpha (ER*α*) isoform and that this might be independent of the hypothalamus-pituitary-adrenal axis (Roesch 2006; Wegorzewska et al. 2008).

As expected, creatinine clearance (a proxy for glomerular filtration rate) decreased with age (Fig.3) across all groups. Although not inevitable, the decrease in glomerular filtration rate with age is well documented (Corman and Michel 1986; Joles and Poston 2010). The GFR decline begins at around age 30–40 in humans and affects both males and females. There are studies suggesting that the decline accelerates after about age 65–70, particularly in women (Botev et al. 2009). Similarly in female rats, regardless of strain, the kidneys’ efficiency to filter blood falls with age (Corman and Michel 1986) and renal damage progresses more slowly in females compared to males or ovariectomized females (Sasser et al. 2012).

We observed that fetal exposure to maternal low-protein diet worsened albuminuria (Fig.3) and proteinuria (Table2). Since albumin is filtered by the glomeruli and it is reabsorbed by the proximal tubular cells during endocytosis, its presence in the urine is an indication not only of the tubular transport but also of function of the glomeruli (Birn and Christensen 2006; Gorriz and Martinez-Castelao 2012). It has been shown that females of certain mouse strains such as C57BL6 are resistant to development of glomerulosclerosis until they reach menopause (Zheng et al. 2004). It has also been shown that estrogen blunts development of glomerulosclerosis by regulating genes involved in extracellular matrix (ECM) turnover in a manner leading to the prevention of ECM accumulation in the mesangium. This was accelerated by ovariectomy (Potier et al. 2001; Elliot et al. 2003). This is in agreement with our studies that provide a comprehensive overview of aging kidney in presence and absence of estrogen underlying the important role that estrogen might play in the progression of renal disease. The oldest rats analyzed, at 18 months, showed the largest increase in albuminuria (Fig.3).

The changes triggered by maternal low-protein diet are also present at the level of gene expression, transcription factors, and protein expression. Studies on aging glomeruli in calorie restricted Fischer 344 rats revealed that changes in gene expression of different cell types in glomeruli concerned genes with regulatory regions that possess binding domains for NF-kappaB. The transcription factor NF-kappaB activates age-related transcriptional changes in the glomerulus such as ceruloplasmin or nephrin (Wiggins et al. 2010).

NF-kappaB has been associated with proinflammatory and fibrotic processes in the kidney (Zheng et al. 2004; Wiggins et al. 2010). We have observed that the CD3^+^ glomerular infiltration was much higher in the absence of female hormones (Fig.6). The CD3 antigen is a marker of T cells, which are responsible for the cell-mediated immunity (Mazairac and Joles 2010). Presence of CD3^+^ cells suggests activation of phagocytes, antigen-specific cytotoxic T-lymphocytes or release of various cytokines in kidney (Colen and Jolesz 2010; Dendooven et al. 2011). The signs of inflammatory reaction in the kidney were associated with increased NFkB expression in the ovariectomized rats that was not related to age (Fig.6). The role of NFkB in controlling T-cell development has been reviewed recently (Gerondakis et al. 2014).

Exposure to a MLP diet has been shown to impact on the expression of the components of the RAS (Woods et al. 2001; Sahajpal and Ashton 2003; McMullen and Langley-Evans 2005b). This has been suggested to mediate some of the long-term effects of maternal protein restriction on tissue structure and physiological function in the offspring. MLP offspring exhibit a decrease in renin mRNA and decrease in Ang II level in kidneys up to at least day 5 post birth (Woods et al. 2001). This suggests that from a very early age MLP offspring show malfunction in the expression and actions of RAS components. Early treatment with drugs directed at the RAS can prevent the development of high blood pressure in MLP exposed offspring (Sherman and Langley-Evans 1998). Early administration of angiotensin-converting enzyme inhibitor captopril, prevents the development of hypertension programmed by intrauterine exposure to a maternal low-protein diet in the rat (Sherman and Langley-Evans 1998, 2000). Antihypertensive treatment in early postnatal life modulates prenatal dietary influences upon blood pressure, in the rat (Sherman and Langley-Evans 2000). In this study, we focused only on the AGTR2 protein expression as its expression has been associated with protective effects against female aging and it might be consider as a putative therapeutic target. A more detailed analysis of RAS components in the future could provide more complex view on the Ang II involvement in accelerated female aging.

The AGTR2 is expressed in the rat kidney (Miyata et al. 1999; Sahajpal and Ashton 2003; McMullen and Langley-Evans 2005b) and its expression has been shown to be affected by MLP diet (Sahajpal and Ashton 2003, 2005; McMullen and Langley-Evans 2005a,b). Under our experimental conditions we found that expression AGTR2 changed with age (Fig.5). AGTR2 was expressed at a higher level at 18 months of age, in the MLP group. The AGTR2 is believed to act in a protective manner in the presence of estrogen (Antus et al. 2003; Macova et al. 2008). The greater expression of AGTR2 at 18 months of age in the MLP group may be related to an aging-related loss of the protective ostrogen role, and was associated with an increase in oxidative damage in the LP group at 18 months, (Fig.5).

Mean arterial blood pressure was significantly lower in the intact control females in compare to MLP groups and ovariectomized females (Fig.4). Estradiol replacement in MLP offspring females has been shown to reverse the effect of the ovariectomy but not the influence of MLP on blood pressure (Bongartz et al. 2010). Maternal diet manipulation might affect some other blood pressure regulatory pathways such as RAS and that Estradiol replacement cannot normalize. Angiotensin II acting via AGTR2 can lower blood pressure by acting directly on vascular dilation or via activation of protein dephosphorylation, the NO-cGMP system and phospholipase A, which in turn mediates release of arachidonic acid (Gerritsen et al. 2010). NO has been recognized to play an important role in the regulation of blood pressure. NO synthesis has been recently related to the ER*α* presence in the kidney which is affected by estrogen levels (Bongartz et al. 2010). It has been shown that total NO decreases in chronic kidney diseases, in aging male but not in aging female rats (Baylis 2009). Oxidative stress, which has been associated with aging, affects NO production, for example reactive oxygen species decrease NO by increasing asymmetric dimethylarginine levels and inactivation of NO (Baylis 2012). Carbonyl levels showed age-diet-related increases in oxidative stress in the kidney in the maternal LP group. Higher concentrations of kidney carbonyls might reduce local NO production and in turn affect the AGTR2 receptor signaling pathway.

## Conclusions

In conclusion, maternal low-protein diet accelerates kidney damage particularly in the absence of female hormones. This investigation provide basis for further analysis of AGTR2 which might act via interaction with female hormones to protect against aging across species in females.
